# Central s-resistin deficiency ameliorates hypothalamic inflammation and increases whole body insulin sensitivity

**DOI:** 10.1038/s41598-018-22255-3

**Published:** 2018-03-02

**Authors:** María Rodríguez, Cristina Pintado, Eduardo Moltó, Nilda Gallardo, Carmen M. Fernández-Martos, Virginia López, Antonio Andrés, Carmen Arribas

**Affiliations:** 1 Biochemistry Section, Faculty of Biochemistry and Environmental Sciences and Regional Centre for Biomedical Research, UCLM, Avda de Carlos III s/n, 45071 Toledo, Spain; 2 Biochemistry Section, Faculty of Science and Chemical Technologies and Regional Centre for Biomedical Research, UCLM, Avda de Camilo José Cela 10, 13071 Ciudad Real, Spain; 3 Wicking Dementia Research and Education Centre, University of Tasmania. Medical Science Precint. 17 Liverpool St Hobart, Tasmania, 7000 Australia

## Abstract

S-resistin, a non-secretable resistin isoform, acts as an intracrine factor that regulates adipocyte maduration, inflammatory and insulin response in 3T3-L1 cells. However, its intracellular function *in vivo* is still unknown. In this study, we analyze the central role of s-resistin, decreasing its hypothalamic expression using an intracerebroventricular injection of lentiviral RNAi. The data present herein support an improvement in the hypothalamic leptin and insulin signaling pathway upon s-resistin downregulation. Furthermore, hypothalamic levels of pro-inflammatory markers decrease, meanwhile those of the anti-inflammatory cytokine IL-10 increases. Interestingly, peripheral NEFA decreases alike circulating leptin and resistin levels. These data demonstrate that hypothalamic s-resistin controls fuel mobilization and adipokines secretion. Importantly, central s-resistin downregulation improves systemic insulin sensitivity, as demonstrated after an IPGTT. Interestingly, our data also indicate that s-resistin downregulation could improve hypothalamic inflammation in aged Wistar rats. Altogether, our findings suggest that hypothalamic s-resistin seems to be a key regulator of the brain-fat axis which links inflammation with metabolic homeostasis.

## Introduction

In the last years, the population affected by obesity, metabolic syndrome, type 2 diabetes and inflammatory diseases have increased dramatically, mainly in developed countries, being considered a worldwide epidemic. Thus, control and prevention of these noncommunicable diseases represent one of the great challenges of modern society. Numerous studies have been carried out to elucidate the molecular mechanisms underlying the development of metabolic and inflammatory diseases^1,2^. However, pathogenesis of many of them remains unknown.

It is known that hypothalamus integrates hormonal and metabolic signals to control the overall body homeostasis^1,3^. Many of these signals are circulating hormones and cytokines secreted by adipocytes, such as leptin, resistin or tumor necrosis factor alpha (TNF-α), which act as systemic signals modulating energy metabolism and insulin signalling pathways in peripheral tissues^3,4^. Besides, crossing the blood-brain barrier, adipocytokines exerted potent effects on specific groups of neurons in the hypothalamus^4,5^, where are involved in the neuroendocrine regulation of food intake, energy expenditure^3^ and the inflammatory response^6,7^. The imbalance of this regulatory adipo-hypothalamic axis promotes obesity and inflammatory disorders, which contribute to the onset of type 2 diabetes.

Resistin was early described in rodents as an adipocytokine that affects whole body energy metabolism, impairs insulin sensitivity and adipocyte differentiation, linking obesity and type 2 diabetes mellitus^8,9^. Mice lacking resistin show low blood glucose levels after fasting and a decreased expression of gluconeogenic enzymes^10^ whereas high levels of circulating resistin cause insulin resistance in liver, muscle and adipose tissue, leading to glucose intolerance^11,12^. Furthermore, resistin also regulates the inflammatory response, suggesting that inflammation could mediate its inhibitory effects on insulin action and adipogenesis^13^. In addition to its direct actions on peripheral organs, several studies show that resistin may play a role regulating the adipose-hypothalamic axis^14–16^, like other circulating hormones such as leptin or insulin, among others^5,17^. In this regard, central resistin treatment in rodents increases negative modulators of the insulin pathway in the hypothalamus, affecting glucose and lipid metabolism^15,16^. Furthermore, central resistin treatment induces hepatic insulin resistance^18^.

In Wistar rats, our group demonstrated the existence of a new short resistin isoform, s-resistin, in the white adipose tissue (WAT)^19^. This isoform is generated by alternative splicing (10–15% of resistin expression) and lacks the secretory peptide signal, suggesting a role as intracellular protein. In fact, s-resistin is mainly located in the nucleus of 3T3-L1 cells^19^. Interestingly, the expression of resistin isoforms is differentially regulated in WAT by ageing, fat depot or nutritional status, suggesting that alternative splicing plays a role in this regulation^20^.

To date, our knowledge about the biological function of s-resistin comes from *in vitro* studies performed in 3T3-L1 cells expressing both s-resistin and resistin sequences. These results showed that s-resistin, like resistin, impaired insulin action in adipocytes^21^ and inhibits adipocyte differentiation by stimulation of the expression of pro-inflammatory molecules, such as TNF-α and interleukin 6 (IL-6)^22^. Interestingly, s-resistin significantly increased the expression and secretion of TNF-α at early stages of adipocyte differentiation^22^. All of these observations suggest that s-resistin functions extends the possibilities of resistin actions, and could act as an intracrine factor that regulates the adipocyte maturation, the inflammatory state and the cellular insulin response. However, the *in vivo* intracellular function of s-resistin and its role in rat physiology remain unknown.

In this work, we demonstrate for the first time that the short nuclear isoform, s-resistin, is highly expressed in the hypothalamus of Wistar rats, suggesting a specialized function for s-resistin in that tissue. Remarkably, the mRNA levels of this resistin variant are increased in the hypothalamus of aged rats, confirming that alternative splicing plays a key role in the regulation of resistin isoforms expression during ageing^20^. Since, resistin acts through peripheral and central pathways promoting insulin resistance and affecting overall energy homeostasis^10,16^, we decided to analyse the role of central s-resistin in whole-body physiology. To that end, we knocked down central s-resistin expression in 3-month-old Wistar rats by an intracerebroventricular (ICV) injection of RNAi against s-resistin mRNA.

Unexpectedly, the results presented herein show that deficiency in hypothalamic s-resistin expression ameliorates central inflammation and improves hypothalamic insulin signaling pathway. Moreover, central s-resistin downregulation significantly changes the expression of key neuropeptides implicated in the regulation of food intake and energy expenditure, and improved whole body insulin sensitivity. Besides, the results obtained in 24-months-old rats treated ICV with RNAi-s-resistin showed several responses to hypothalamic s-resistin knockdown, although they were less pronounced than those observed in young-rats. Taken together, these data indicate that s-resistin isoform could play an important role in the molecular alterations underlying central and systemic insulin resistance and support the hypothesis that central s-resistin could be an astounding player in the interplay between inflammation and metabolism.

## Results

### S-resistin is widely expressed in the hypothalamus and its levels are increased with ageing

**Figure 1:**
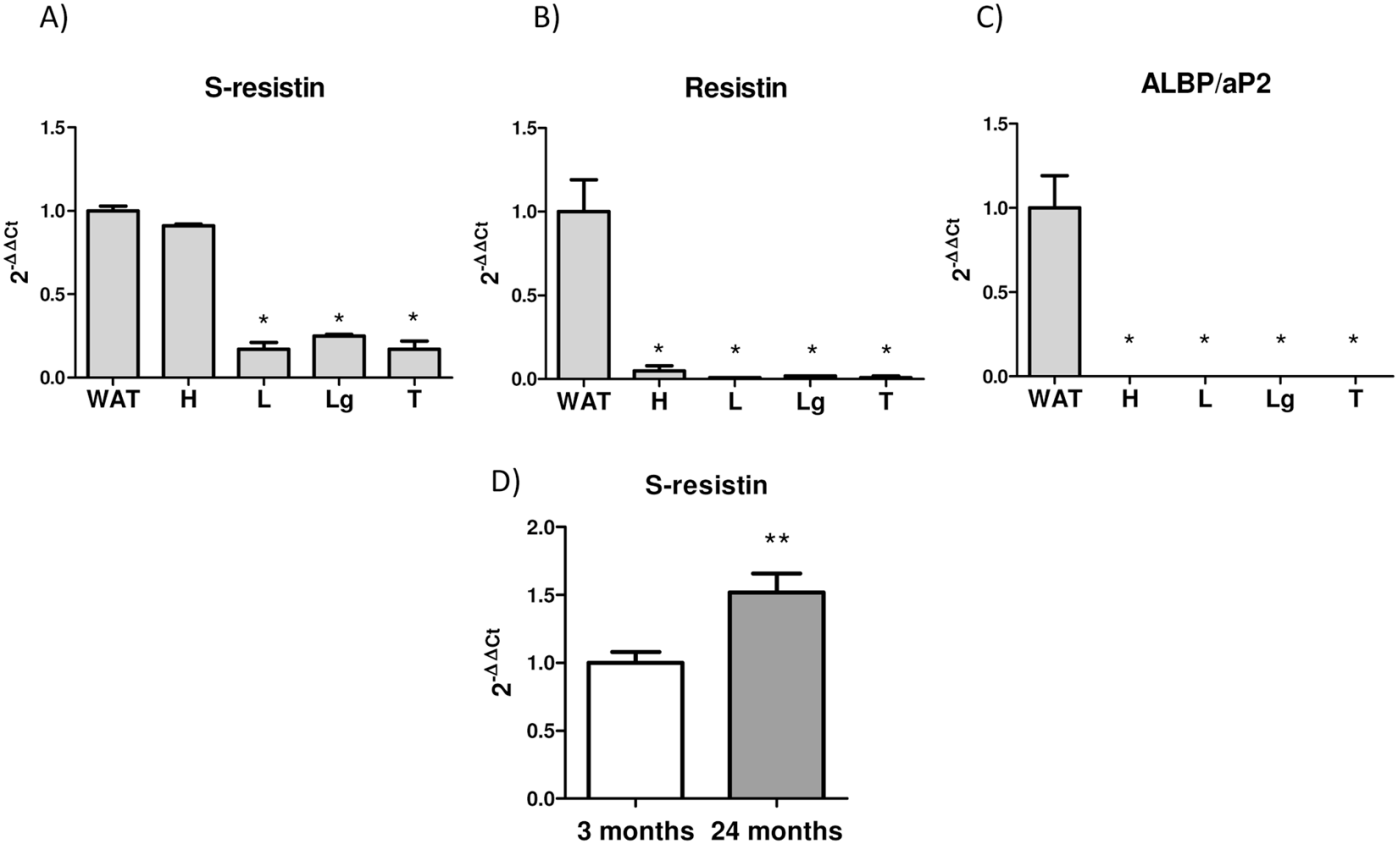
S-resistin expression is high in the hypothalamus and increases with ageing. The expression of s-resistin (**A**), resistin (**B**) and ALBP/aP2 (**C**) in different tissues of 3-months old Wistar rats was determinated by qPCR using SYBRgreen or Taqman depending on the gene (see Methods section). 18S rRNA was used as endogenous control. Values are the means ± SEM; n = 4 separated determinations per group of animals, each sample made in duplicate. White adipose tissue (WAT), hypothalamus (H), liver (L), lung (Lg) and testicle (T), *p < 0,01 vs WAT (one-way ANOVA followed by Bonferroni test). Values were normalized to 1 versus WAT. (**D**) Hypothalamic expression of s-resistin in 24-month-aged compared to 3-month-young Wistar rats analyzed by qPCR. Values are the means ± SEM; n = 4 independent experiments per group. *p < 0,05 compare to young (Student’s t-test).

Resistin is mainly expressed in WAT and, in a lesser extent, in other tissues of rat and mouse^23^. However, the tissue distribution of the short resistin isoform mRNA is not known. Here, using a probe to distinguish the two resistin isoforms, we demonstrate for the first time that, although at different expression levels, s-resistin is present in all the rat tissues analyzed and, as expected, their highest expression is detected in WAT (Fig. 1A). However, in contrast to the long isoform (Fig. 1B), s-resistin is notoriously abundant in the hypothalamus, where the expression levels are similar to those observed in the WAT (Fig. 1A). In order to rule out any contamination of the sample with WAT, the expression of adipocyte lipid binding protein (ALBP/aP2) was analyzed. As shown in Fig. 1C, ALBP/aP2 is expressed exclusively in this tissue. Therefore, our data demonstrate that s-resistin is the main resistin isoform expressed in hypothalamus of Wistar rats. Interestingly, in pre-diabetic aged Wistar rats with central leptin and insulin resistance^24,25^, s-resistin expression is increased in the hypothalamus (Fig. 1D).

Based on these data we hypothesized that s-resistin could have an important role in the hypothalamic function and in the development of central and peripheral insulin resistance.

### Downregulation of central s-resistin levels modify central appetite-regulating neuropeptides and improves peripheral insulin sensitivity

**Figure 2:**
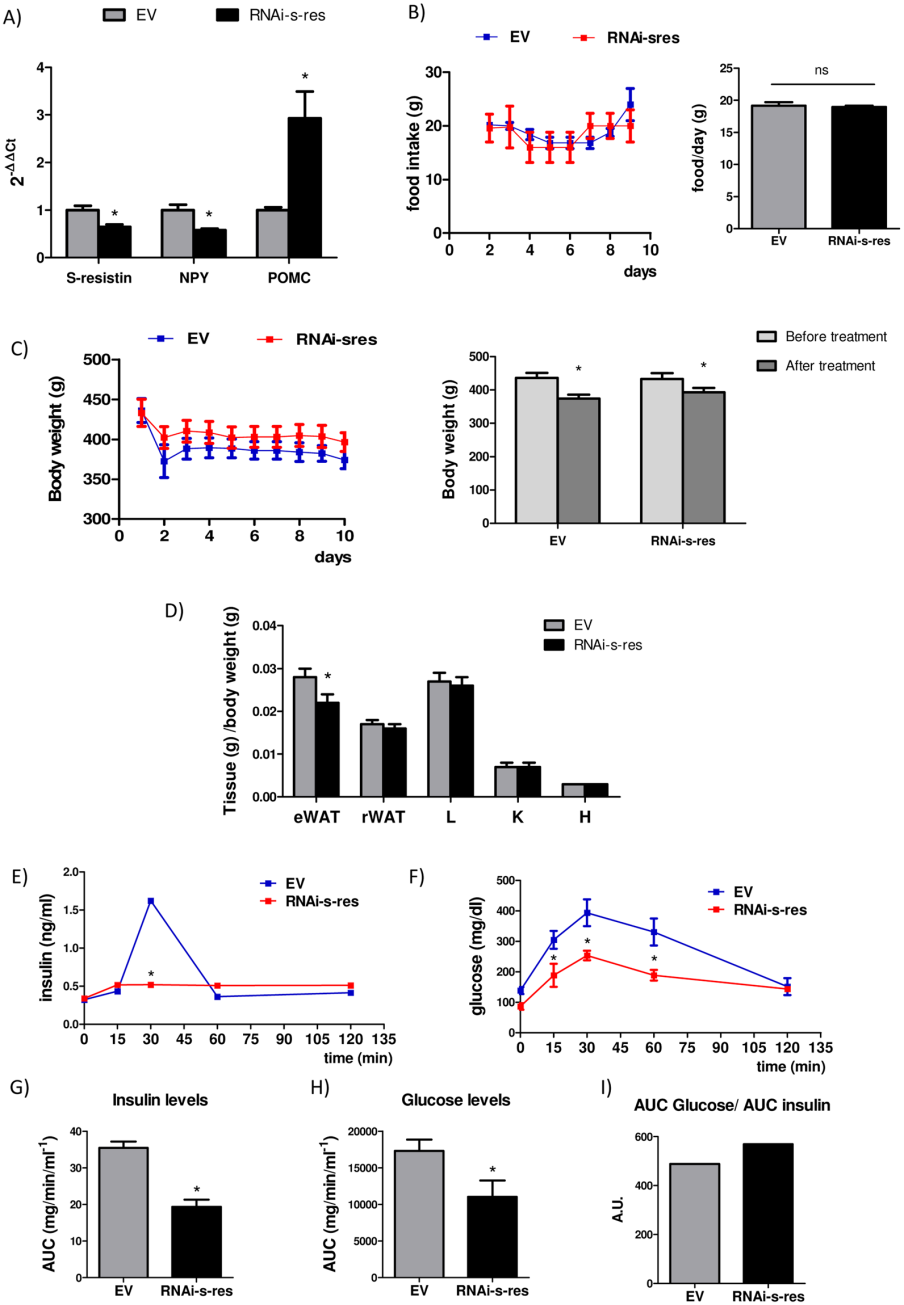
LV-RNAi-s-res administration modifies central appetite-regulating neuropeptides, decreases the weight of epididymal adipose tissue and improves peripheral insulin sensitivity. (**A**) Hypothalamic expression of s-resistin, NPY and POMC after ICV treatment analyzed by qPCR. (**B**) Daily food intake (g) and food intake average along the experiment (g/day). (**C**) Daily body weight (g) and body weight before and after the treatment in control and injected with s-resistin RNAi. (**D**) Tissue weights expressed as tissue weight (g)/body weight (g). Epididymal white adipose tissue (eWAT), retroperitoneal white adipose tissue (rWAT) liver (L), kidney (K) and heart (H). (**E**,**F**) **IPGTT:** Each animal was intraperitoneal injected with 2 g/kg of glucose 40%. Blood samples are taken from the tale vein at different times point and insulin (**G**) and glucose plasma levels (**H**) were measured. Animals treated with the s-resistin RNAi showed an improvement in the glucose tolerance and an increase in the global insulin sensibility as indicate the ratio AUC glucose/AUC insulin (**I**). Values are the mean ± SEM; n = 4–5 separated determinations per group of animals, each sample made in duplicate. *p < 0,05 compared to EV (Student’s t-test). Animal injected with the empty virus (EV), Animal injected with virus with s-resistin RNAi (RNAi-s-res).

In order to elucidate the role of hypothalamic s-resistin in whole body physiology, we knocked down hypothalamic s-resistin in 3-month-old Wistar rats by an ICV injection of RNAi against s-resistin mRNA (RNAi-s-res) (See Methods section, Supplementary Table S1, supplementary Fig. S1). The results showed in Fig. 2A indicate that in treated animals with RNAi the expression of s-resistin decreased by 35% compared to control animals infected with empty virus. Besides, we did not find significant variation in the food intake of RNAi-s-res treated rats compared to the control (Fig. 2B). Even though, animals lost weight seems to be due to the surgery itself rather than to the s-resistin RNAi injection (Fig. 2C). Moreover, as shown in Fig. 2D, the ratio epididymal WAT weight/ body weight significantly decreased in treated animals when compared to controls.

Surprisingly, although there was no variation in food intake, we found that the mRNA levels of NPY decreased (Fig. 2A) whereas those of the anorexigenic pro-opiomelanocortin (POMC) increased (Fig. 2A) in rats with a lesser amount of hypothalamic s-resistin. These results support the idea that s-resistin may participate somehow in this hypothalamic neurocircuits but without affecting food intake.

In order to determine the influence of central s-resistin deficiency on the whole body insulin sensitivity, we carried out an intraperitoneal glucose tolerance test (IPGTT) in these animals, after a week of the ICV intervention. Our results show that animals treated with RNAi-s-res are more sensitive to insulin (Fig. 2E), and have an improved glucose tolerance compared to control animals (Fig. 2F). Furthermore, we estimated the area under the curve (AUC) of insulin and glucose levels to calculate the glucose AUC/ insulin AUC ratio as an indicator of the overall sensitivity to insulin (Fig. 2E–I). As it is shown in Fig. 2I, a hypothalamic decrease of s-resistin expression is accompanied by an increase in this ratio in treated animals indicating a significant improvement in insulin sensitivity in RNAi-s-res infused animals.

On the other hand, as shown in Table 1, the animals with diminished hypothalamic s-resistin expression have lower plasma leptin, non-esterified fatty acids (NEFA) and resistin levels, indicating that interruption of s-resistin action at central level regulate fat mobilization. Besides, this treatment decreased plasma insulin without affecting fasting glucose levels. Thus, this data support that the hypothalamic s-resistin downregulation leads to an improvement in systemic insulin sensitivity. In fact, when HOMA-IR index was calculated a significant decrease was observed in ICV RNAi-s-res treated animals (12 ± 2 vs 6, 1 ± 0.5; Table 1). The values measure of serum cytokines show an increase in the levels of anti-inflammatory IL-4 in rats with knockdown central s-resistin expression (Table 1). All these data point to an improvement in overall insulin sensitivity and glucose homeostasis in RNAi-s-res treated rats.

**Table 1:** Level of plasma metabolic parameters of used animals.

	EV	RNAi-s-res
NEFA (mmol/L)	1.24 ± 0.02	1.13 ± 0.03^*^
KB (mmol/L)	0.18 ± 0.03	0.16 ± 0.03
TAG (mg/dl)	118 ± 9	106 ± 9
Glucose (mg/dl)	106 ± 8	117 ± 5
Lactate (mmol/L)	3.8 ± 0.1	4.2 ± 0.1
Insulin (ng/ml)	1.8 ± 0.5	0.84 ± 0.1^*^
Resistin (ng/ml)	15 ± 1	10 ± 2^*^
Leptin (ng/ml)	4.8 ± 0.3	3.7 ± 0.6^*^
HOMA-IR	12 ± 2	6.1 ± 0.5^*^
IL-4 (pg/ml)	67 ± 3	86 ± 3^*^
IL-10 (pg/ml)	192 ± 40	222 ± 56
IL-1β (pg/ml)	36 ± 10	31 ± 8
IL-6 (pg/ml)	1469 ± 249	1375 ± 95
TNF-α (pg/ml)	3 ± 1	3 ± 1
IFN-γ (pg/ml)	1204 ± 149	857 ± 429

Animals injected with the empty virus (EV) and with virus with s-resistin RNAi (RNAi-s-res). Values are the means ± SEM; n = 4–5 independent experiments per group. Biochemical determinations were made in duplicate. *p ≤ 0,05 compared to EV (Student’s t-test). NEFA: non-esterified fatty acid; KB: ketonic bodies; TAG: triacylglycerides; HOMA-IR: Homeostasis model assessment for insulin resistance.; IL: Interleukine; TNF: Tumor Necrosis Factor; IFN-γ: Interferon gamma.

### Central downregulation of s-resistin improves hypothalamic insulin and leptin response

**Figure 3:**
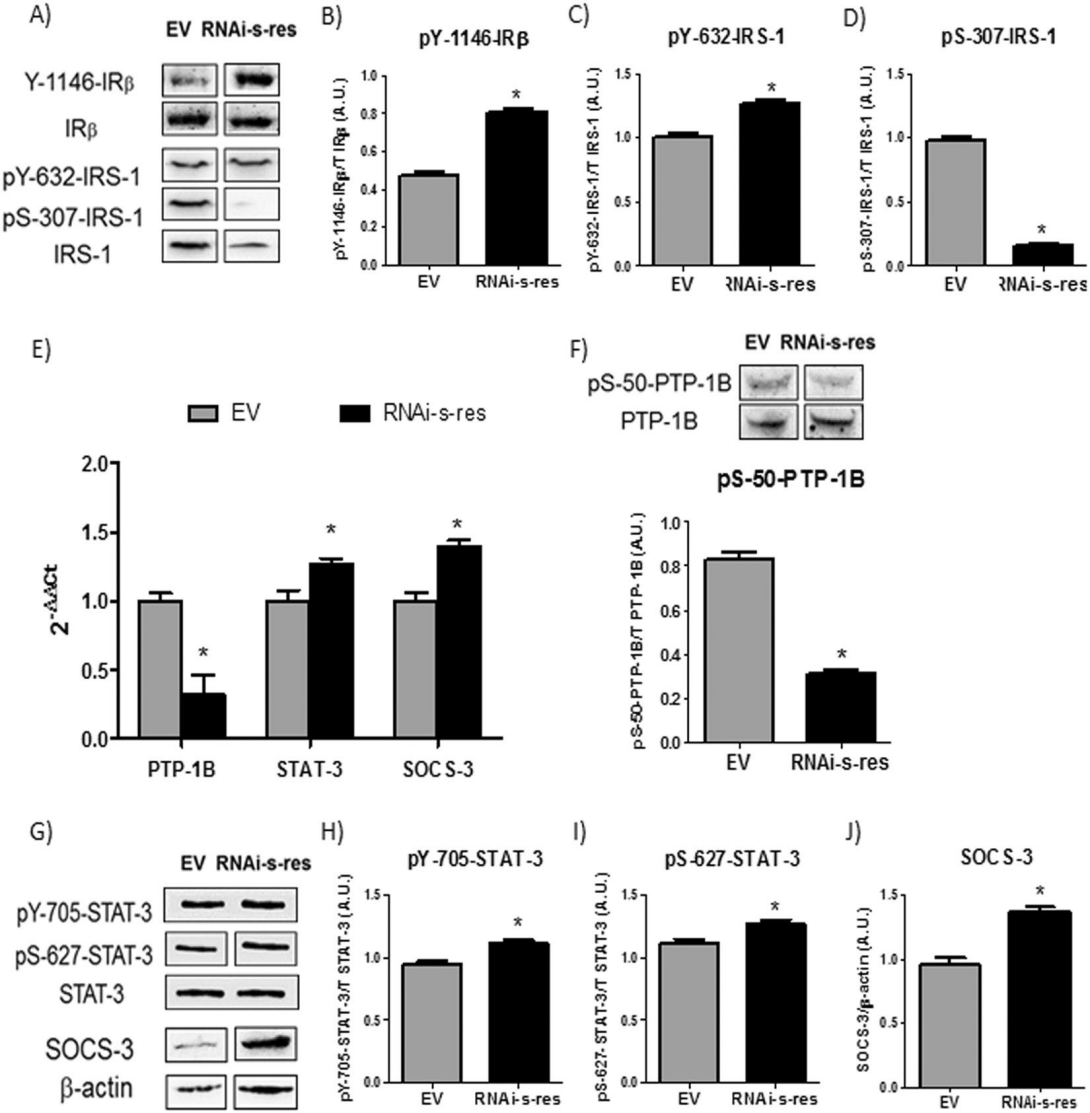
Downregulation of central s-resistin expression improves central insulin and leptin pathway. (**A**) Representative western blot (WB) of protein and Y-1146 phosphorylation of IRβ, IRS-1 protein and Y-632 and S-307 phosphorylation of IRS-1. (**B**) pY-1146-IRβ/IRβ ratio (**C**) pY-632-IRS-1/IRS-1 ratio and (**D**) pS-307-IRS-1/IRS-1 ratio determined by optical density analysis. (**E**) Expression levels of PTP-1B, STAT-3 and SOCS-3 after ICV treatment analyzed by qPCR. (**F**) Representative WB of protein and S-50 phosphorylation of PTP-1B and pS-50-PTP-1B/PTP-1B ratio determined by optical density analysis. (**G**) Representative WB of protein and Y-705 and S-627 phosphorylation of STAT-3 and SOCS-3 protein. (**H**) pY-705-STAT-3/STAT-3 ratio (**I)** pS-627-STAT-3/STAT-3 ratio and (**J)** SOCS-3/β-actin ratio determined by optical density analysis. Values are the means ± SEM; n = 4–5 separated determinations per group of animals, each sample made in duplicate, *p ≤ 0,05 compared to EV (Student’s t-test). Animal injected with the empty virus (EV), Animal injected with virus with s-resistin RNAi (RNAi-s-res). The bands of gels/blots were cropped from different parts of the same gel. Full-length blots are presented in Supplementary Figures S3, S4 and S5.

Next, we analyzed the mRNA, protein mass and the phosphorylation levels of several intermediaries of insulin and leptin signaling pathway. Figure 3 shows that central s-resistin downregulation increased basal Tyr phosphorylation of both IR and IRS-1 (Fig. 3A–C), while decreased Ser307 phosphorylation of IRS-1 (Fig. 3A,D) in the hypothalamus. In addition, while PTP-1B mRNA and phosphorylation levels were decreased (Fig. 3E,F), the expression of SOCS-3 was augmented and STAT-3 mRNA and phosphorylation levels were also increased (Fig. 3E,G–J). These data support an improvement in the insulin and leptin signaling pathways in the hypothalamus upon s-resistin downregulation.

### Downregulation of central s-resistin levels reduces the inflammatory state in the hypothalamus

**Figure 4:**
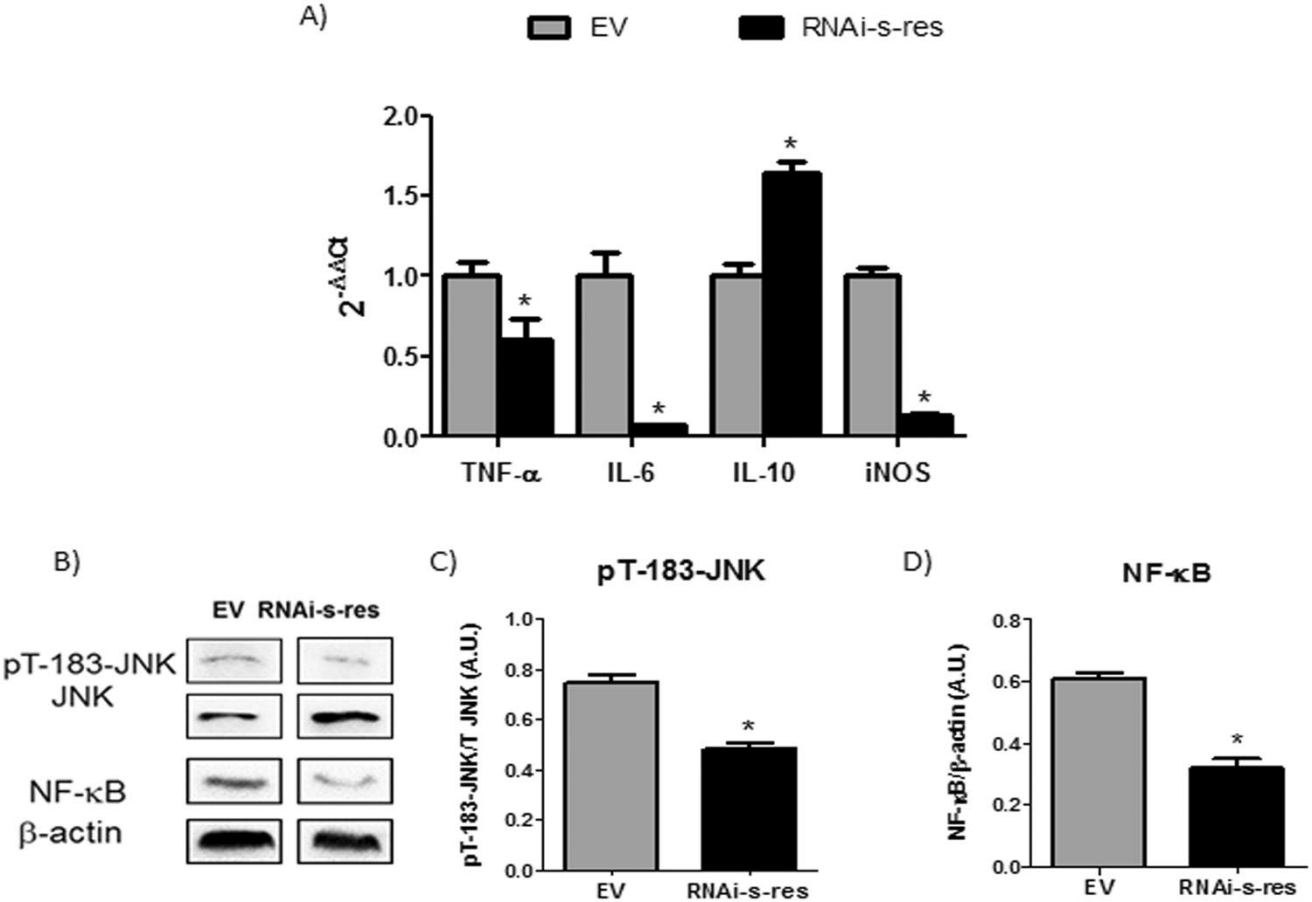
Downregulation of s-resistin expression levels in the hypothalamus decreases the inflammation status. (**A**) Hypothalamic expression levels of TNF-α, IL-6, IL-10 and iNOS in ICV treated animals analyzed by qPCR. (**B**) Representative WB of protein and T-183 phosphorylation of JNK and NF-κB protein (**C**) pT-183-JNK/JNK and (**D**) NF-κB/β-actin ratio determined by optical density analysis. Values are the means ± SEM; n = 4–5 separated determinations per group of animals, each sample made in duplicate, *p ≤ 0,05 compared to EV (Student’s t-test). Animal injected with the empty virus (EV), Animal injected with virus with s-resistin RNAi (RNAi-s-res). The bands of gels/blots were cropped from different parts of the same gel. Full-length blots are presented in Supplementary Fig. S6.

Inflammation in the hypothalamus may cause central leptin and insulin resistance and deregulation of the energy balance^26,27^. Therefore, we decided to measure the expression levels of different inflammation-related signalling molecules such as cytokines TNF-α, IL-6, IL-10 and the inducible nitric oxide synthase (iNOS) in control and RNAi-s-res treated animals. Likewise, the protein levels of necrosis factor-κB (NF-κB) and basal Thr phosphorylation levels of janus kinase (JNK) were analyzed. As shown in Fig. 4(A–D), downregulation of s-resistin reduced the expression of hypothalamic pro-inflammatory markers TNF-α, IL-6 and iNOS as well as the Thr phosphorylation levels of JNK and protein levels of NF-κB. Conversely, the levels of the anti-inflammatory cytokine IL-10 increased in RNAi treated animals (Fig. 4A). All these results, suggest that the improved insulin signaling in hypothalamus of animals treated with RNAi-s-res could be due to a decrease in the hypothalamic inflammatory response.

### Central downregulation of s-resistin expression in 24-months-old animals increases POMC expression but do not improve peripheral insulin sensitivity

**Figure 5:**
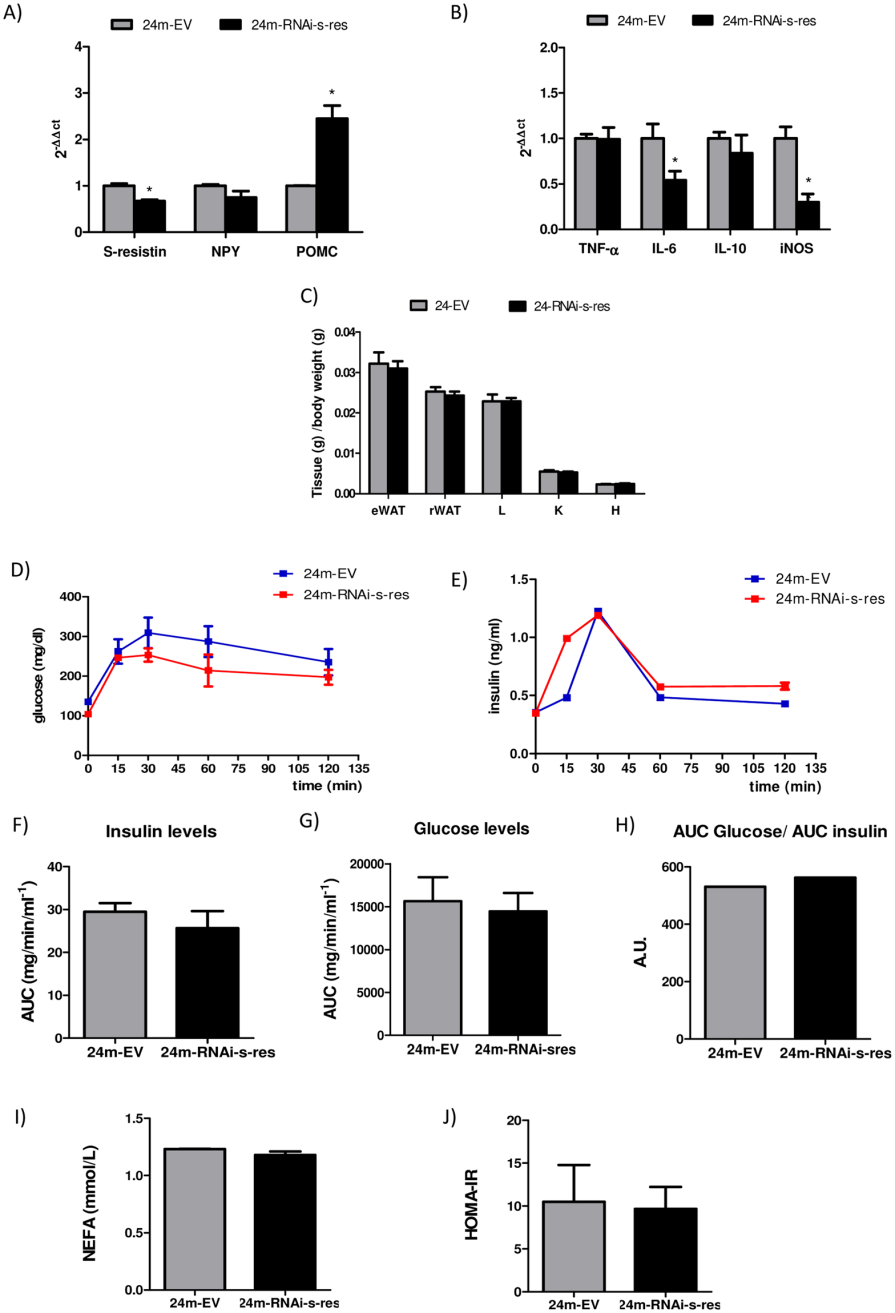
LV-RNAi-s-res administration in 24-months-old animals do not improve peripheral insulin sensitivity. (**A**) Hypothalamic expression of s-resistin, NPY and POMC after ICV treatment analyzed by qPCR. (**B**) Hypothalamic expression levels of TNF-α, IL-6, IL-10 and iNOS in ICV treated animals analyzed by qPCR. (**C**) Tissue weights expressed as tissue weight (g)/body weight (g). Epididymal white adipose tissue (eWAT), retroperitoneal white adipose tissue (rWAT) liver (L), kidney (K) and heart (H). (**D,E**) **IPGTT:** Each animal was intraperitoneal injected with  2g/kg of glucose 40%. Blood samples are taken from the tale vein at different times point and insulin (**F**) and glucose plasma levels (**G**) were measured. RNAi-s-res old treated animals do not ameliorate its response to IPGTT, and no significate differences were observed in the ratio AUC glucose/AUC insulin (**H**). (**I**) NEFA serum levels in 24-months-old animals after treatment ICV with EV or RNAi-s-res (**J**) HOMA-IR index calculated for old treated animals. Values are the mean ± SEM; n = 4–5 separated determinations per group of animals, each sample made in duplicate. *p < 0,05 compared to EV (Student’s t-test). 24-months animal injected with the empty virus (24 m-EV), 24-months animal injected with virus with s-resistin RNAi (24 m-RNAi-s-res).

Aged Wistar rats showed central and peripheral inflammation and leptin and insulin resistance^24,25,28^. Likewise, these animals presented high hypothalamic s-resistin expression (Fig. 1D). Therefore, and to analyze the influence of central s-resistin on the development of insulin resistance with ageing, we decided to knockdown central s-resistin levels in 24-months-old Wistar rats. As shown in Fig. 5A, aged rats infused with RNAi-s-res showed an increase of central POMC expression, without changes in the food intake and body weight parameters (Supplementary Fig. S2A,B), as it was observed in young ICV RNAi-s-res treated animals (Fig. 2B,C). Nevertheless, no changes were observed in the transcript levels of NPY in these animals. Notably, although there were no significant differences in the expression of TNF-α and IL-10 in old rats, we appreciate that lentivirus injection reduced the expression of iNOS and IL-6, two inflammatory factors that lies downstream of TNF-α pathway, although the effect was less pronounced that those observed in young rats (Fig. 5B).

Furthermore, in aged rats, lentivirus injection did not decrease the weight of eWAT (Fig. 5C), nor the circulating levels of glucose, insulin, resistin (Supplementary Fig. S2C,D) and NEFA (Fig. 5I). Similarly, the IPGTT response and the HOMA-IR values were not modified when central s-resistin expression was downregulated in aged rats (Fig. 5D–H,J). These data indicate that in rats with high hypothalamic levels of inflammation and insulin resistance, central reduction of s-resistin expression at the levels obtained in this work, partially reverses hypothalamic basal inflammation without improving systemic insulin sensitivity.

## Discussion

Here we report novel data potentially relevant to central nervous system (CNS) control of insulin resistance. In addition to the importance of insulin-mediated responses in peripheral target tissues, CNS insulin signalling regulates energy and glucose homeostasis by acting on hypothalamic neurocircuits^29^. However, the molecular mechanism by which the hypothalamic neurons sense the energy status is not well understood. In this regard, the hormone resistin, acting through the CNS, has been demonstrated to promote both peripheral and hypothalamic insulin resistance, impairing energy homeostasis^16,18^. Here, we show that the intracellular resistin spliced variant, s-resistin, is highly expressed in hypothalamus of Wistar rats. Together, our results indicate that s-resistin is a physiological component of the metabolic network required for the regulation of glucose homeostasis. The reduction of central s-resistin expression in 3-months-old Wistar rats, improves hypothalamic insulin and leptin signaling as well as peripheral insulin sensitivity, as demonstrated after an IPGTT. These animals showed higher glucose tolerance and an increased glucose AUC/insulin AUC ratio, as well as a decrease in the HOMA-IR index. Taken together, these parameters reflect an improvement in overall insulin sensitivity when the central s-resistin expression is down-regulated.

Hypothalamic inflammation, which may result from different situations such as ageing, over-nutrition or disease, is tightly associated with feeding and metabolic dysregulations^6^. It is known that TNF-α induces the expression of PTP-1B, at least in part by transactivation of NF-κB, nevertheless, PTP-1B is not considered to be involved in the onset, but rather in the continued deterioration of insulin and leptin action in the brain^30^. Our results indicate that disruption of endogenous s-resistin plays an important role in the control of hypothalamic inflammation. In this sense, we found a markedly reduction in the hypothalamic IL-6, TNF-α mRNA and NF-κB protein levels in 3-month-old RNAi treated rats. Besides, central downregulation of s-resistin lead a decrease in PTP-1B mRNA expression as well as protein activation. In agreement, rats treated with s-resistin RNAi showed increased basal Tyr phosphorylation of both IR and IRS-1. Furthermore, the inhibitory phosphorylation of IRS-1 on Ser307 decreased in the treated rat hypothalamus. This serine phosphorylation can be induced by many serine kinases, but JNK is a central point in this process^31^. In accord, Thr phosphorylation levels of JNK were decreased in the RNAi-s-res treated rats. Hence, we hypothesized that s-resistin could have an essential role in the hypothalamic function and in the development of central insulin resistance throughout modulation of the inflammatory response.

Additionally, induction of NF-κB, C/EBPβ and pro-inflammatory cytokines probably mediated by resistin binding to the TLR4 receptor, is a feature of resistin treatment in several biological models^22,32,33^. Indeed, Benomar *et al*.^16^ reported the direct binding of resistin to TLR4 in the hypothalamus, leading to the activation of the associated proinflammatory pathways. However, it has been previously suggested that endogenous ligands may trigger TLRs during tissue injury and certain disease states, which could promote inflammation in absence of infection^34^. Hence, it would be interesting to consider the possibility that s-resistin might participate in the intracellular TLR arrangements of the early response to injury in the hypothalamus.

IL-10 has been reported to inhibit the production of cytokines such as TNF-α and IL-6 in glial cells *in vivo* and *in vitro*^35^ and that this cytokine regulates the expression of iNOS at the transcriptional level, reducing NO production by inhibiting NF-κB-dependent transcription of genes^36^. In addition, it has been demonstrated that IL-10 expression in the CNS increases during recovery from brain inflammation, suggesting that IL-10 in CNS is required for inflammatory state remission^37^. In our work, the anti-inflammatory cytokine IL-10 mRNA increased, whereas iNOS expression was downregulated after the ICV treatment, supporting the anti-inflammatory effect of reducing s-resistin levels in the brain.

On the other hand, Ropelle *et al*.^26^ describes that IL-10 and IL-6 engage receptors that recruit janus kinases and activate predominantly STAT-3 in the hypothalamus, promoting a re-balance in the energy intake in obese animals. So, our results further suggest that IL-10 might be involved in STAT-3 activation and SOCS-3 upregulation observed in the hypothalamus of RNAi-s-res rats.

Leptin signalling in certain hypothalamic nuclei induces the expression of POMC and activates STAT-3^38^. Also, resistin have been implicated in this regulation. Thus, central infusion of recombinant human resistin in rats leads upregulation of the POMC and downregulation of the NPY gene expression in the arcuate nucleus and a markedly decrease in both, food intake and body weight^16^. Here, we show that knockdown central s-resistin, significantly reduced the expression of NPY while induced the expression of POMC in the hypothalamus, without alterations in food intake and body weight. Thus, our results confirm that the interruption of central s-resistin action increase hypotalamic leptin signaling as demonstrate by the higher phosphorylation levels of STAT-3. This is in agreement with the marked reduction in the circulating levels of NEFA, resistin and leptin observed in RNAi treated rats, whose high levels were positively correlated with adiposity, insulin resistance, diabetes and metabolic syndrome^39,40^. Thus, we can consider that the levels of these plasma factors which reach the hypothalamus significantly fall in s-resistin knockdown rats, when compared to the control rats. Therefore, all these results point out to an improvement of central and peripheral energy metabolism and leptin sensitivity and suggest that central s-resistin could control peripheral fat mobilization as well as cytokines secretion. In this sense, we observed an increase of serum levels of the anti-inflammatory cytokine IL-4 even though we did not find variations in other circulating cytokines.

Additionally, we reported previously that epididymal WAT is the most resistin-sensitive adipose tissue^20^, and interestingly, only this tissue decreases its weight in 3-month-old rats treated with RNAi-s-res, suggesting that central s-resistin could participate more specifically regulating the resistin adipo-hypothalamic axis.

Here, we also found that s-resistin expression is increased in 24-month-old Wistar rats, which have been considered as a pre-diabetic model with central leptin and insulin resistance^24,25^ accompanied by peripheral insulin resistance, hyperlipidemia and increased adiposity^41^. Furthermore, old Wistar rats showed hypothalamic inflammation, with increased transcript levels of TNF-α, CD11b, MCP-1 and TLR4 in the hypothalamus^28^. Thus, in order to know the effects of s-resistin knockdown under these metabolic and inflammatory state, we knocked down central s-resistin expression in 24-months-old rats. In this sense, the decrease of s-resistin expression obtained in aged rats with the same virus title used in young rats (33% *vs* 35% in young animals), was not enough to downregulate hypothalamic s-resistin levels to match the ones observed in young animals. Nevertheless, we observed 150% increase in the hypothalamic expression levels of POMC (*vs* 300% in young animals), while IL-6 and iNOS were decreased by a 50% and 35% respectively (*vs* 90% and 80% respectively in young animals), although lentivirus injections in aged rats did not improve insulin sensitivity and glucose tolerance, as assessed by intraperitoneal glucose tolerance test and HOMA-IR measure. These data suggest that knockdown of s-resistin in old rats could improve hypothalamic inflammation.

**Figure 6:**
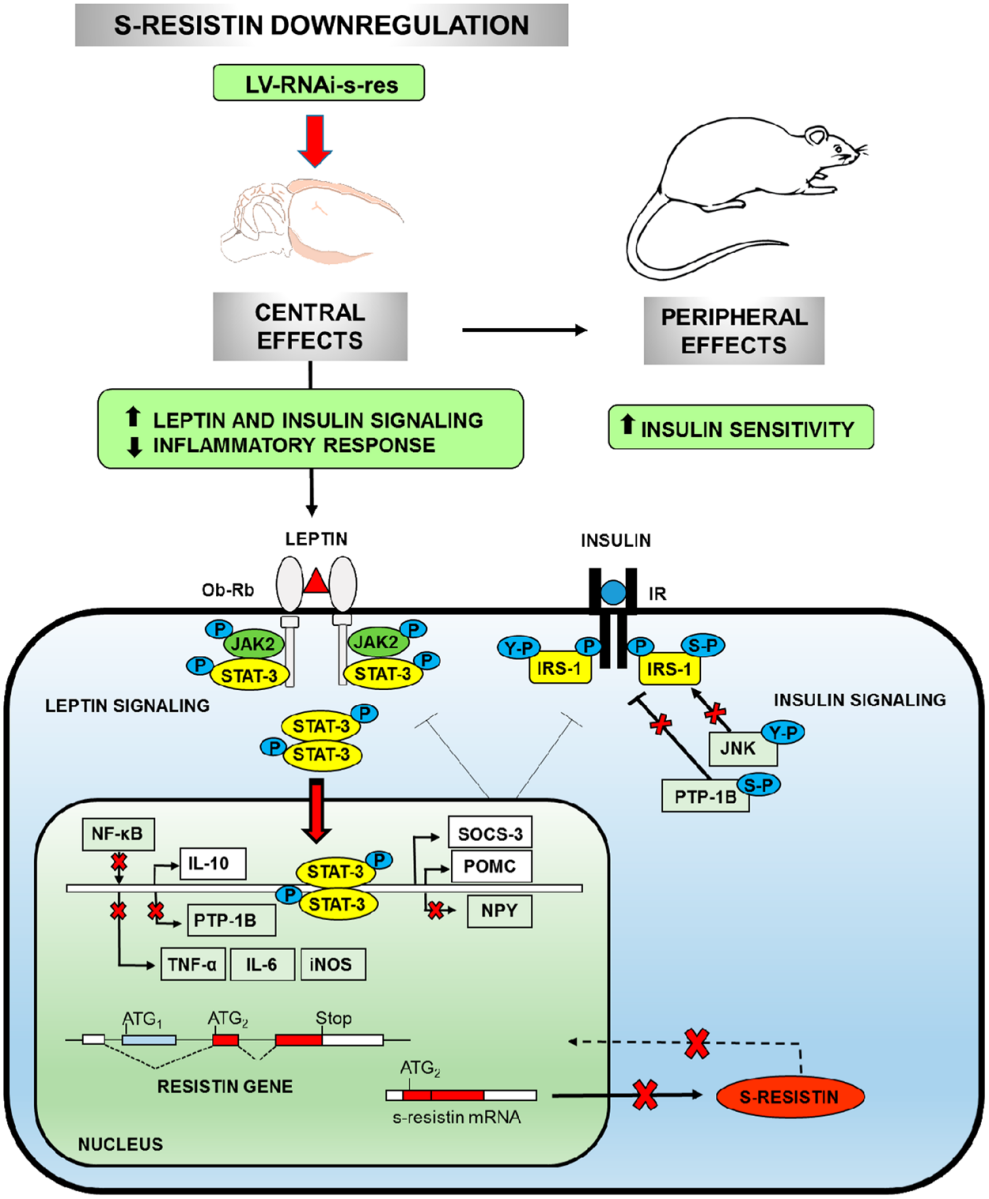
This figure summarizes the central s-resistin downregulation effects on hypothalamic and peripheral insulin and leptin sensitivity. Knocked down central s-resistin by ICV injection of lentiviral RNAi, improves hypothalamic insulin pathway increasing IRβ and IRS-1 activity by the up-regulation in tyrosine phosphorylation. In agreement with that, the inhibitory Ser307-phosphorylation of IRS-1 decreased in treated rats together with the JNK activity, major responsible of IRS-1 Ser-phosphorylation. The improvement of central insulin signalling is also supported by the fall of PTP-1B expression and protein activation, possibly due to the improvement of the inflammatory state (see below). Besides, the hypothalamus raises its leptin sensibility by increasing STAT-3 activation that promotes POMC expression and prevent the NPY one. The downregulation of TNFα, IL-6, iNOS, JNK and the transcription factor NK-κB combined with the upregulation of IL-10 support a decrease on the inflammatory status in the hypothalamus of treated rats. All these improvements in the central inflammatory state contribute equally to ameliorate the hypothalamic insulin and leptin signalling. Also, the reduction of central s-resistin decreased adipokines secretion and enhanced peripheral insulin sensitivity. Take together all these results indicate that s-resistin could be a key player implicated in the development of central insulin resistance and inflammatory disease.

Hypothalamic neurons play a crucial role in the control and maintenance of glucose homeostasis and fuel expenditure throughout the body, sensing the peripheral hormones and nutrients. Since s-resistin is an intracellular isoform, it is possible that the hypothalamic responses to these circulating signals could involve the molecular regulation of alternative splicing process in resistin gene, modulating the intracellular s-resistin levels. Thus, s-resistin could acts as an intracrine hypothalamic sensor linking peripheral signals with both, hypothalamic insulin and leptin responsiveness and inflammatory status (Fig. 6). Therefore, the understanding of s-resistin functions could contribute to elucidate the role of resistin isoforms in metabolic and inflammatory diseases. Further studies will be required to understand the physiological significance of s-resistin in CNS and to establish an adequate association between hypothalamic s-resistin levels, inflammation and altered metabolic homeostasis in aged animals.

## Methods

### RNAi assays in cells

KH1-LV was the lentivirus used. This LV carried the transgene for enhanced green fluorescent protein (EGFP) and for ampicillin resistance. Total plasmid DNA from LV was isolated using QIAfilter Plasmid Purification Midiprep Kit (Qiagen, Hilden, Germany) following the manufacturer’s instructions. After digesting with *Sma*I and *Xba*I, the digested plasmid was purified using ISOLATE Plasmid Mini Kit (Bioline) following the manufacturer’s instructions. Two target sequences (Supplementary Table S1) for Wistar rat mRNA were chosen according to Katahdin’s siRNA design on line tool. All primers were supplied by Life Technologies and there are located in position 28 and 180 of s-resistin (S1 and S2) Finally, S2/AS2 primers were used.

Murine 3T3-L1-s-res pre-adipocytes were previously obtained in our laboratory^22^, HEK 293 FT and HeLa cells were grown in Dulbecco’s modified Eagle’s medium (DMEM) containing 10% foetal bovine serum (FBS) (Gibco BRL) and 100 U/ml penicillin (Gibco) and 100 mg/ml streptomycin (Gibco) at 37 °C in a 5% CO_2_ atmosphere.

3T3-L1-s-res cells were treated with different combinations of RNAi using Lipofectamine® 2000 Reagent (Life Technologies), following the manufacturer’s instructions. Cells without transfecting and transfected with KH1-LV without RNAi were used as negative controls. The cells were harvested 48 h after transfection for expression analysis to prove the efficiency of the inhibition. Construction 2 was chosen because a reduction of 74% of s-resistin mRNA levels was observed when 3T3-L1-s-res were transfected with it (Supplementary Fig. S1A).

### Lentivirus production and packaging for *in vivo* assays

Lentiviral particles were produced by transfecting HEK 298 FT cells seeded in 10 cm dishes with 10 µg of vector DNA together with two helper plasmids (pCMV and pMD) using Ca_3_(PO_4_)_2_ precipitation according to published protocols^42^. The virus-containing cell culture supernatants were harvested 48 h after transfection and concentrated by ultracentrifugation at 30000 rpm in a SW-41 rotor for 90 min. The virus pellet was subsequently resuspended in 100 µl of the supernatant and titrated by serial dilutions on HeLa cells. The virus preparation used for the infections of the animals had a title of 10^8^ tu/ml.

### Animals

Male 3 and 24-months-old Wistar rats were individually housed and maintained on a 12 h light/dark cycle at a controlled temperature (20–25 °C) and humidity (50%) with free access to food and water. All animal experiments were conducted according to the European Union laws (2010/63/EU) and following the Spanish regulations (RD 53/2013) for the use of laboratory animals. The experimental protocols were approved by the institutional and regional ethical committees (University of Castilla-La Mancha). All efforts were made to minimize animal suffering and to reduce the number of animals used.

#### Lentivirus injection

3 and 24-months rats were anesthetized by inhalation of a mixture of O_2_ and isoflurane and placed on the stereotaxic apparatus (David Koppf, Tujunga, CA). Rats were injected with lentivirus containing the antisense sequence selective for s-resistin chosen (RNAi-s-res) or control empty virus (EV) into the left lateral ventricle using the following coordinates, according to Paxinos’ atlas: AP: −0,8 mm; L: 1,6 mm; DV: −3,4 mm^43^. An opening in the skull was made with a dental drill. The viral stock (10^8^tu/ml) was injected at a speed of 1 μl/min with a 10 μl Hamilton syringe. The needle was left in place for additional 5 min to avoid reflux along the injection track. Total volume injected was 5 μl. Rats were returned to the housing colony and sacrificed by decapitation after LV administration. LV used carried the transgene for enhanced green fluorescent protein (EGFP). Therefore, the presence of green fluorescence signal in the hypothalamus was tested to confirm that the lentivirus injected through the lateral ventricle was able to infect hypothalamic cells (supplementary Fig. 1B). Two period of recovery from surgery were tested, 10 and 20 days respectively. Brains were rapidly removed and hypothalamus were dissected, frozen in liquid nitrogen and stored at −70 °C until use. Retroperitoneal (rWAT) and epididymal (eWAT) fat pads, liver, heart and kidneys were dissected, weighed and flash frozen in liquid nitrogen and stored at −70 °C until use. Also, blood samples were centrifuged and plasma was frozen until use. Finally, 10 days after surgery was chosen because the disruption of s-resistin expression was more efficient at this time point (Supplementary Fig. S1C). Moreover, we analyzed the impact of s-resistin downregulation in overall insulin sensibility. We also found better results after 10 days post-surgery (Supplementary Fig. S1D).

#### Plasma metabolites, hormones and cytokines analysis

Lactate and glucose were measured in blood using an Accutrend analyzer (Roche). Plasma Triacylglyceride (TAG) levels were determined by an enzymatic kit from Biosystem (Barcelona, Spain). NEFA and ketone bodies (KB) were measured with enzymatic kits from WAKO Chemical (Neuss, Germany). Plasma leptin and insulin levels were assayed using specific rat ELISA kits from SPI-Bio (Montigny le Bretonneux, France). Plasma resistin was assessed using a rat resistin ELISA kit (BioVendor, Brno, Czech Republic), following the manufacturer’s instructions. Cytokines were analysed using MILLIPLEX MAP Rat Cytokine/Chemokine Magnetic Bead Panel, and Immunology Multiplex Assay form Merk-Millipore (Darmstadt, Germany). Homeostasis model assessment for insulin resistance (HOMA-IR) was calculated as fasting insulin (µU/ml) × fasting glucose ([mmol/liter]/22.5) as described earlier^41^.

#### Intraperitoneal glucose tolerance test (IPGTT)

Overnight fasted rats were administered 40% glucose solution intraperitoneally (2 g glucose/kg of body weight) two days before sacrifice. Blood samples were taken from the tail vein before the glucose load (*t* = 0) and 15, 30, 60, and 120 min after glucose administration. Blood glucose was determined immediately using an Accutrend Glucose Analyser (Roche). Blood samples were centrifuged and plasma was frozen at −70 °C until insulin estimation. Overall changes in glucose and insulin during IPGTT were calculated as the area under the curve above the basal level (Glucose and Insulin AUC respectively). The ratio of Glucose AUC to Insulin AUC was used as an index of whole body insulin sensitivity^41^.

### RNA and protein isolation

Total RNA from, liver, lung and testicle was isolated using RNeasy Mini Kit (Qiagen, Hilden, Germany) and RNA from hypothalamus and adipose tissue was obtained using RNeasy Lipid Tissue Mini Kit (Qiagen, Hilden, Germany) following the manufacturer’s instructions. This procedure allows the isolation of total RNA, DNA and protein fractions from a single sample^44^.

Complementary DNA (cDNA) was synthesized from 1 μg of DNase-treated RNA^45^. Protein pellets obtained using the RNeasy Lipid Tissue Mini Kit (Qiagen, Hilden, Germany),were resuspended in 4% SDS and 8 M urea in 40 mM Tris–HCl. The total recovery and integrity of these fractions were determined by Lowry *et al*.^46^.

### Real time qPCR analysis

Real time quantitative PCR (q-PCR) was performed by using ABI PRISM 7500 Fast Sequence Detection System instrument and software (Applied Biosystem, Foster City, CA). Relative quantification of target cDNA in each sample was performed from 10 ng of cDNA in TaqMan One-Step real time PCR Master Mix and using Pre-Developed TaqMan Assay Reagents (PE Applied Biosystem) for PTP-1B (Rn01640350_g1), SOCS-3 (Rn00585674_s1), STAT-3 (Rn00562562_m1), IL-6 (Rn00561420_m1), IL-10 (Rn00563409_m1), iNOS (Rn00561646_m1), ALBP/aP2 (Rn00670361_m1) and 18 S rRNA (Hs99999901_s1) with VIC as real time reporter was used as control to normalize gene expression. Furthermore, relative quantification of target cDNA in each sample was performed from 10 ng of cDNA in SYBR-Green One-Step real time PCR Master Mix with the following primers supplied by Bonsai Technologies: s-resistin (sense primer, a s-resistin-specific forward primer derived from exon1/exon3 junction^19^: 5′- GAGCTCTCTGCCACGTGCCA-3′; antisense primer 5′-AGTCTATGCTTCCGCACTGGC-3′); resistin (sense primer 5′- AGTTGTGCCCTGCTGAGCTCTCTGCCC-3′; antisense primer 5′- CCCATTGTGTATTTCCAGACCCTC-3′); TNF-α (sense primer 5′- GTGCCTCAGCCTCTTCTCATTCC-3′; antisense primer 5′- GCTCCTCTGCTTGGTGGTTT-3′); NPY (sense primer 5′- CCGCCATGATGCTAGGTAAC-3′; antisense primer 5′- CACCACATGGAAGGGTCTTC-3′); POMC (sense primer 5′- AGCAACCTGCTGGCTTGCAT-3′; antisense primer 5′-CCAGCACTGCTGCTGTTTCT-3′); and rRNA 18S (sense primer 5′-CGGCTACCACATCCAAGGAA-3′; antisense primer 5′-GCTGGAATTACCGCGGCT-3′).

The ΔΔCT method was used to calculate the relative differences between experimental conditions and control groups as fold change in gene expression^47^.

### Western Blot analysis

Proteins were fractionated on a SDS-PAGE gel and blotted for 2 h at 90 V. Western Blot analyses was performed by antibody incubation in PBS containing 0.05% Tween and 5% (w/v) milk powder according to standard protocols.

The following primary antibodies were used in this study: β-actin (Sigma, 1:1000 dilution), SOCS-3 (Santa Cruz, 1:500), pY-1146-IRβ (Sigma, 1:500), IRβ (Santa Cruz, 1:500), pY-632 and pS-307 IRS-1 (SAB Signaling, 1:500), IRS-1 (Cell Signaling, 1:500), pT-183-JNK (Abcam, 1:500), JNK (Abcam, 1:500), Y-705 and S-627 STAT-3 (Cell Signaling, 1:500), STAT-3 (Cell Signaling, 1:500), pS-50-PTP-1B (Abcam, 1:500), PTP-1B (Abcam, 1:500), NF-κB (Abcam, 1:500). Goat anti-rabbit IgG-HRP and rabbit anti-Mouse IgG-HRP (Sigma) were used as secondary antibodies, and ECL Prime (Amersham) reagent was used for developing. Bands were quantified by scanning densitometry with a G-Box densitometer with exposure in the linear range using Gene Tools software (Synergy, Cambridge, UK). The relative levels of phosphorylated and total proteins were normalized to the corresponding amount of total protein mass and β-actin, respectively, in the same sample.

### Statistical analysis

Statistical analysis was performed using one-way ANOVA (GraphPad Prism 5.03 software, GraphPad Software, Inc., San Diego, CA). When the main effect was significant, the Bonferroni post hoc test was applied to determine individual differences between means. Statistical significance between RNAi-s-res treated and control animals was calculated using Student’s t-test. P values less than 0.05 were considered to be statistically significant.

## Electronic supplementary material

Supplementary Information
