# Supplementary material for: Central s-resistin deficiency ameliorates hypothalamic inflammation and increases whole body insulin sensitivity
Source: Sci Rep. 2018 Mar 2;8:3921. doi: 10.1038/s41598-018-22255-3 (PMC5834531; doi:10.1038/s41598-018-22255-3)
Supplement: Supplementary file 1 — Supplementary Information [file 41598_2018_22255_MOESM1_ESM.pdf]

Central s-resistin deficiency ameliorates hypothalamic inflammation and increases whole body insulin sensitivity

María Rodríguez, Cristina Pintado, Eduardo Moltó, Nilda Gallardo, Carmen Fernández-Martos, Virginia López, Antonio Andrés and Carmen Arribas.

INVENTORY OF SUPPLEMENTARY INFORMATION

Supplementary Figure Legend

Supplementary Table S1

Supplementary Figure S1

Supplementary Figure S2

Supplementary Figure S3

Supplementary Figure S4

Supplementary Figure S5

Supplementary Figure S6

SUPPLEMENTARY FIGURE LEGEND

Supplementary Table S1. Data in support of experimental procedures: RNAi assays in cells

Primers used in RNAi design, located in position 28 (S1) and 180 (S2) of s-resistin (AJ555618.1). Sense (S) and antisense (AS). Primers were supplied by Life Technologies.

Supplementary Figure S1. Data in support experimental procedures: RNAi assays in cells and lentivirus injection.

A) RNAi assays in cells. Two RNAi constructs (S1 and S2) were transfected into 3T3-L1-s-res and 3T3-L1-res cell lines, individually or in combination (co-transfection). Expression analysis of s-resistin was determined using qPCR. Clone 2 exhibits the largest decrease of s-resistin expression. WT (without transfect), EV (transfected with empty virus), 1 (transfected with construction 1), 2 (transfected with construction 2) and 1+2 (transfected with a combination of 1 and 2). Values are the means ± SEM; n = 3 independent experiments per group. *p≤ 0,05 compared to EV (one-way ANOVA followed by Bonferroni test). **B) Lentivirus injection:** The LV used carried the transgene for enhanced green fluorescent protein (EGFP). To further confirm the lentivirus reached the hypothalamus by fluorescence microscopy, two animals were transcardially perfused under deep anaesthesia (isofluorane) with 100 mL of PBS containing 10 U/mL heparin followed by 150–200 mL of 4% paraformaldehyde in phosphate buffer, pH 7.4. Brains were removed and then immersed in sucrose in PBS, pH 7.4 until sunken (2–5 days). Tissues were then frozen in isopentane at –20 ºC and 25 µm sections were cut on a

cryostat and mounted on gelatin-coated slides. Finally, sections were examined under a fluorescence microscope (Nikon Eclipse 90i). The presence of green fluorescence signal in the hypothalamus confirms that lentivirus injection through the lateral ventricle is able to infect hypothalamic cells. 3V: Third ventricle; PVN: paraventricular nucleus; Arc: Arcuate nucleus. Scale bar represent 100 μm. **C) S-resistin expression:** Animals were sacrificed 10 days (RNAi-s-res 10 d) or 20 days (RNAi-s-res 20 d) after the operation. S-resistin expression was determined using qPCR. The results indicate that disruption of s-resistin expression was more efficient 10 days after surgery. **D) IPGTT:** Each animal was intraperitoneally injected with 2g/kg of glucose 40%, blood samples are taken from the tale vein at different time points. Overall insulin sensibility was also better after 10 days post-surgery. Values are the means ± SEM; n = 3-5 separate determinations per group of animals, each sample made in duplicate. *p≤ 0,05 compared to EV (Student's t-test).

Supplementary Figure S2. Data support results in Fig. 5. LV-RNAi-s-res administration in 24-months-old animals do not improve peripheral insulin sensitivity.

A) 24-months RNAi-s-res treated animals not shown any change in food intake expressed as food (g) / day. **B**) Animals' body weight decreased after treatment in both control and injected with s-resistin-RNAi. **C)** Fasting glucose levels do not change after treatment in 24-months-old animals. **D)** Insulin and resistin serum levels in 24-months-old animals after treatment ICV with EV or RNAi-s-res. Values are the means ± SEM; n = 3-5 separate determinations per group of animals, each sample made in duplicate. *p≤ 0,05 compared to EV (Student's t-test).

Supplementary Figure S3. Original Western Blots membranes. Data in support Figure 3. Downregulation of central s-resistin expression improves central insulin and leptin pathway.

Western blot of a pool of proteins (40 μg / well) from 5 animals, made in duplicate of (**A**) Y-1146 phosphorylation and total IRβ, (2 minutes of exposition) (**B**) Y-632 and S-307 phosphorylation of IRS-1 and total IRS-1 protein (2 minutes of exposition). All the membranes were cut into the corresponding size of the proteins of interest and re-incubated after stripping with the total antibodies. Each protein (phosphorylated and total form) was detected in the same membrane. Animal injected with the empty virus (EV),

animal injected with virus with s-resistin RNAi (RNAi-s-res). The selected bands appear squared. Uncropped western blot images corresponding to Figure 3A.

Supplementary Figure S4. Original Western Blots membranes. Data in support Figure 3. Downregulation of central s-resistin expression improves central insulin and leptin pathway.

Western blot of a pool of proteins (40 μg / well) from 5 animals, made in duplicate of S-50 phosphorylation and total PTP-1B, (2,5 minutes of exposition). The membrane was re-incubated after stripping with the total antibody. Each protein (phosphorylated and total form) was detected in the same membrane. Animal injected with the empty virus (EV), animal injected with virus with s-resistin RNAi (RNAi-s-res). The selected bands appear squared. Uncropped western blot images corresponding to Figure 3F.

Supplementary Figure S5. Original Western Blots membranes. Data in support Figure 3. Downregulation of central s-resistin expression improves central insulin and leptin pathway.

Western blot of a pool of proteins (40 μg / well) from 5 animals, made in duplicate of (**A**) Y-705 and S-627 phosphorylation and total STAT-3, (1 minute of exposition) and (**B**) SOCS-3 protein (1,5 minutes of exposition) and β-actin (1 minute of exposition). The membrane A was cut into the corresponding size of the proteins of interest and re-incubated after stripping with the total antibody. Each protein (phosphorylated and total form) was detected in the same membrane. SOCS-3 and β-actin were detected in gels done in parallel. Animal injected with the empty virus (EV), animal injected with virus with s-resistin RNAi (RNAi-s-res). The selected bands appear squared. Uncropped western blot images corresponding to Figure 3G.

Supplementary Figure S6. Original Western Blots membranes. Data in support Figure 4. Downregulation of s-resistin expression levels in the hypothalamus decreases the inflammation status.

Western blot of a pool of proteins (40 μg / well) from 5 animals, made in duplicate of (**A**) T-183 phosphorylation and total JNK (2,5 minutes of exposition) and (**B**) NF-κB protein and β-actin (2 minutes of exposition). The membrane A was cut into the corresponding size of the proteins of interest and re-incubated after stripping with the total antibody.

Each protein (phosphorylated and total form) was detected in the same membrane. NF-κB and β-actin were detected in the same membrane incubating with both antibodies at the same time. Animal injected with the empty virus (EV), Animal injected with virus with s-resistin RNAi (RNAi-s-res). The selected bands appear squared. Uncropped western blot images corresponding to Figure 4B.

Primers	Sense primer (5′→3′)	Antisense primer (3′→5′)
S1/AS1	5′-CGTGCCAGCTGCAATGAAGAATTCAAGAGATTC TTCATTGCAGCTGGCACGTTTTTTGT-3′	5′-CTAGACAAAAAACGTGCCAGCTGCAATGA AGAATCTCTTGAATTCTTCATTGCAGCTGGCACG-3′
S2/AS2	5′-ACTGCCAGTGCGGAAGCATAGTTCAAGAGAC TATGCTTCCGCACTGGCAGTTTTTTTGT-3′	5′-CTAGACAAAAAAACTGCCAGTGCGGAAGCAT AGTCTCTTGAACTATGCTTCCGCACTGGCAGT-3′

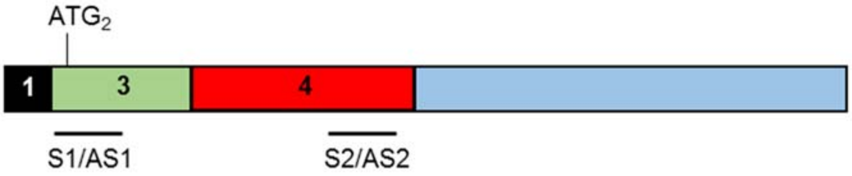

Supplementary Table S1

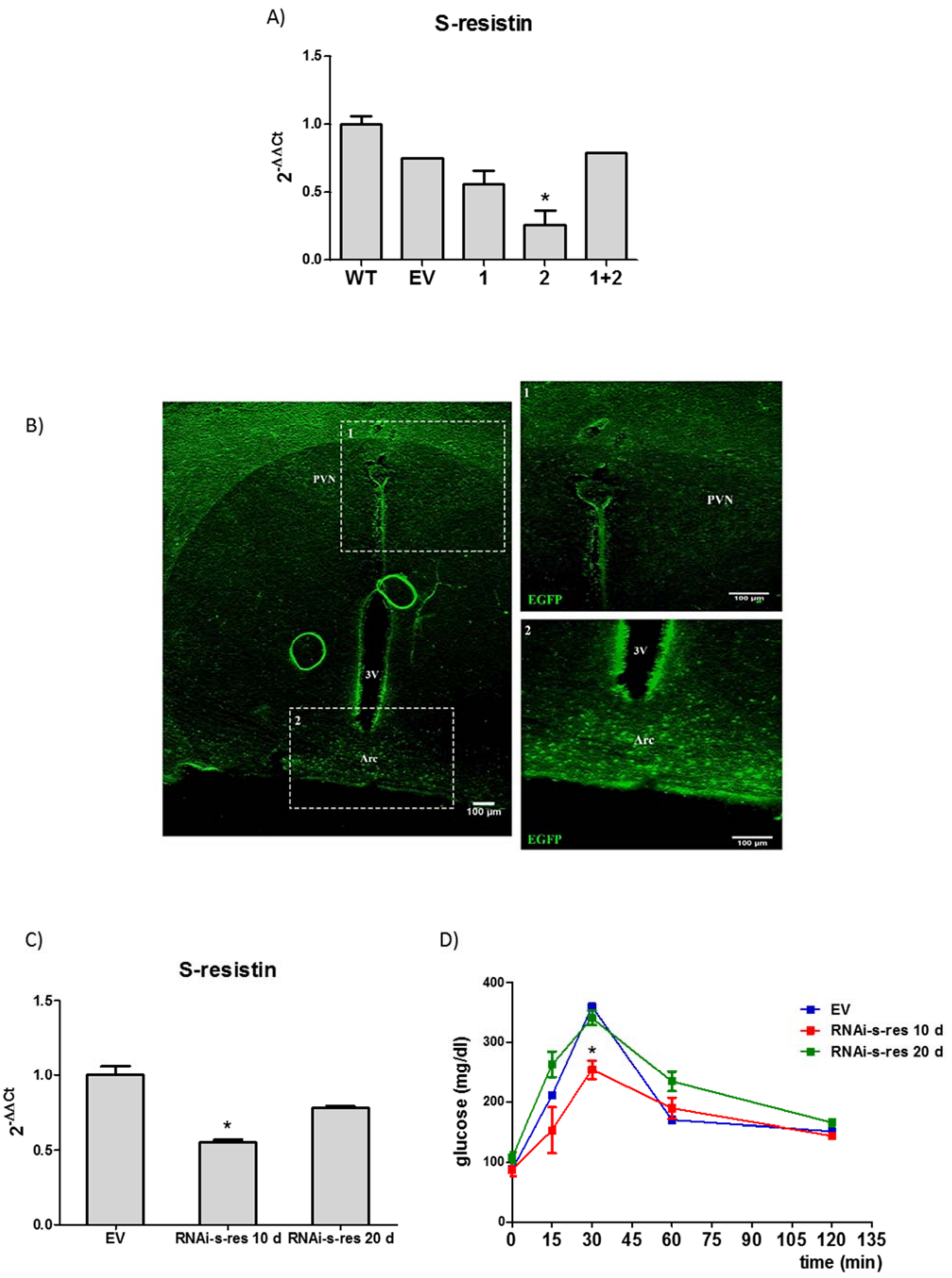

Supplementary Figure S1

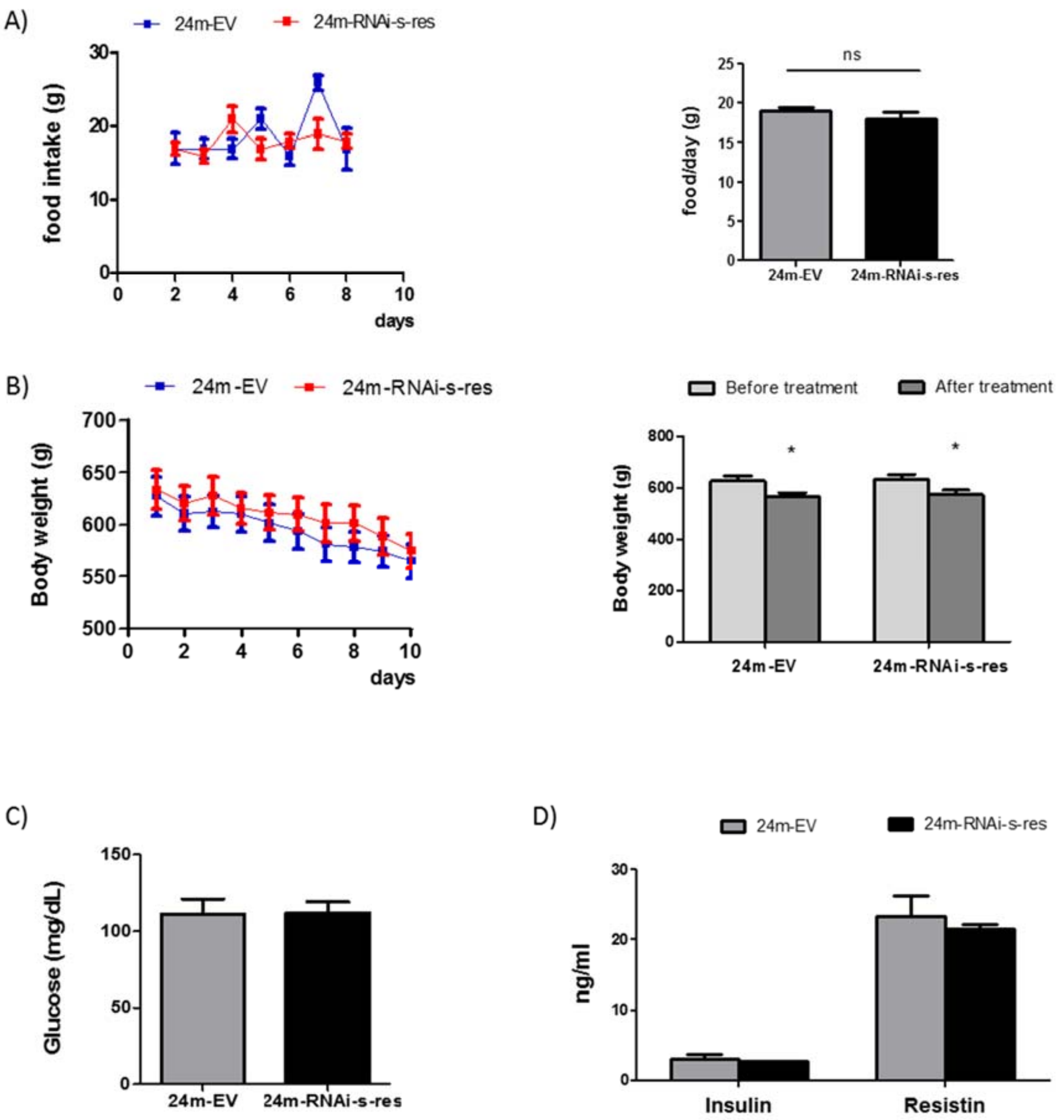

Supplementary Figure S2

Uncropped western blots from Figure 3A

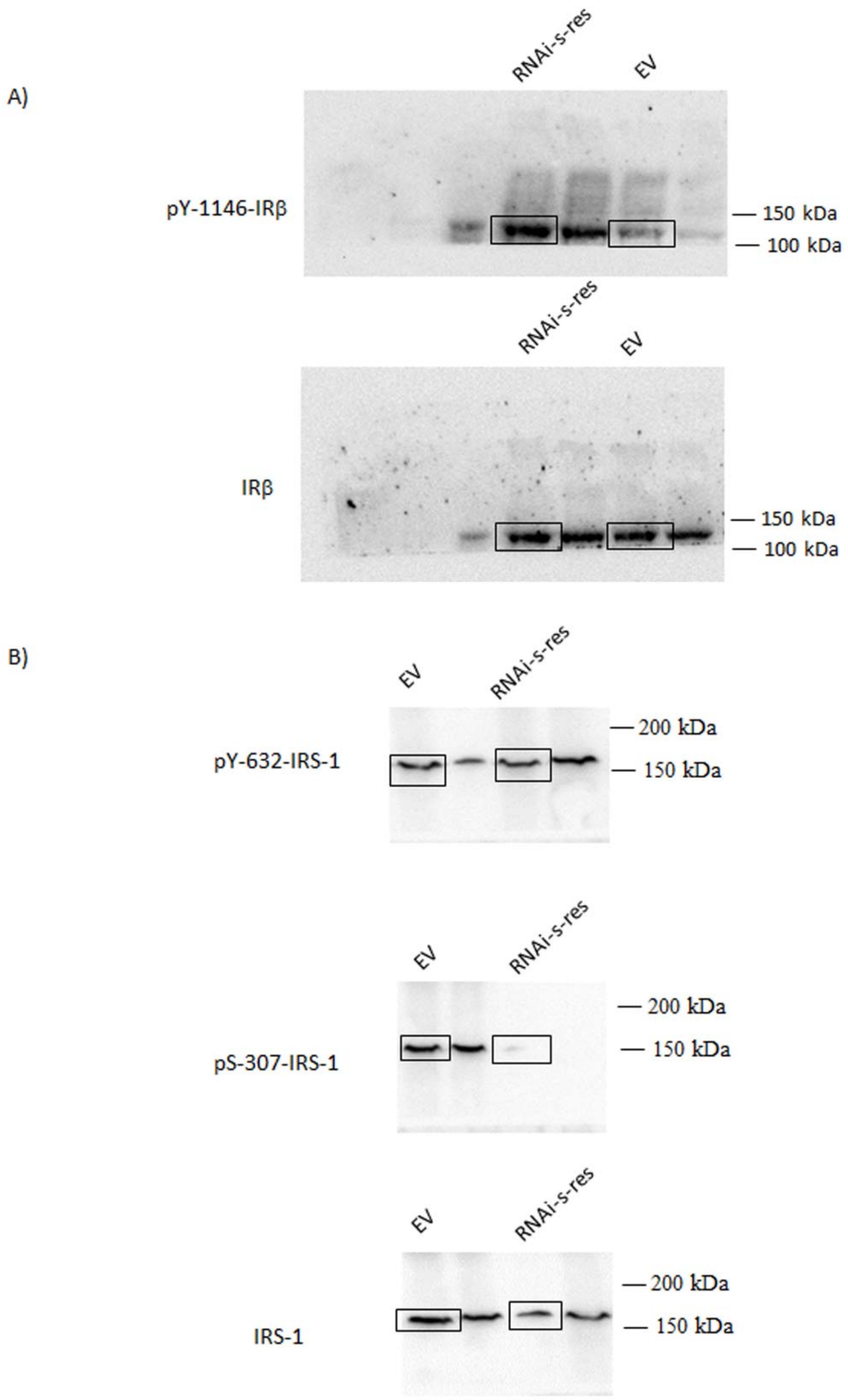

Supplementary Figure S3

Uncropped western blots from Figure 3F

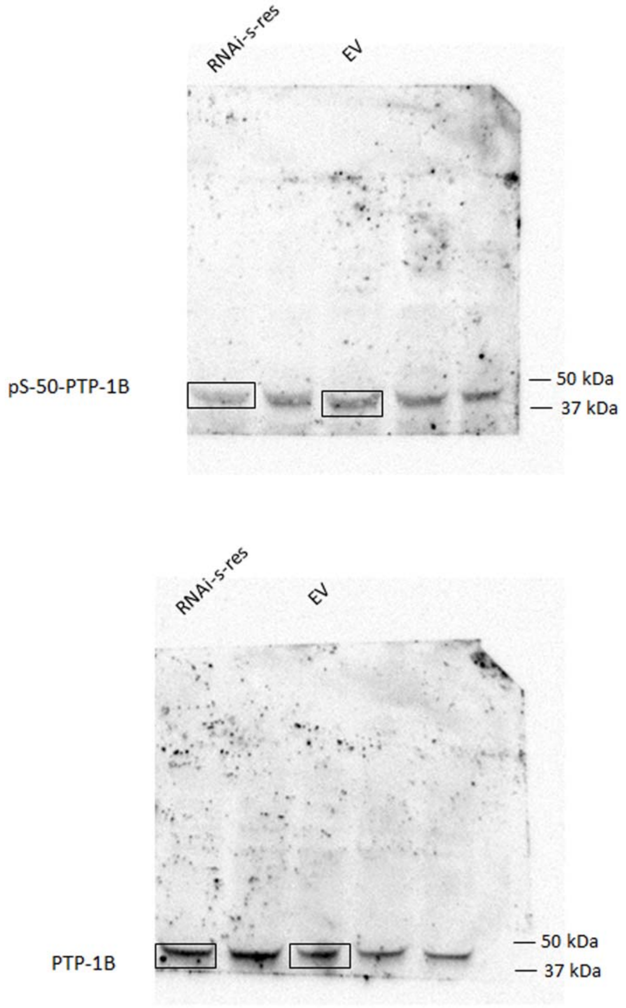

Supplementary Figure S4

Uncropped western blots from Figure 3G

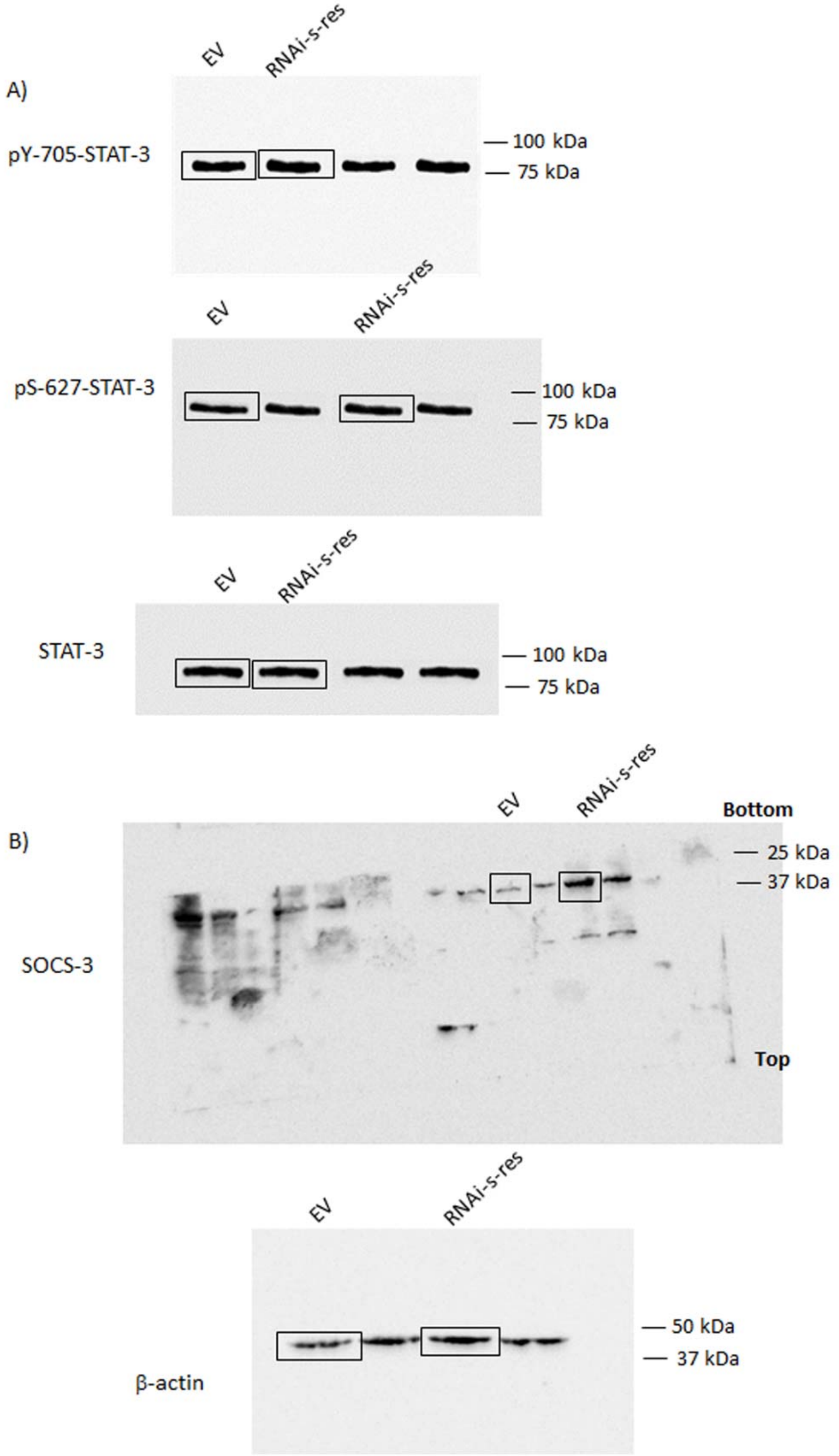

Supplementary Figure S5

Uncropped western blots from Figure 4

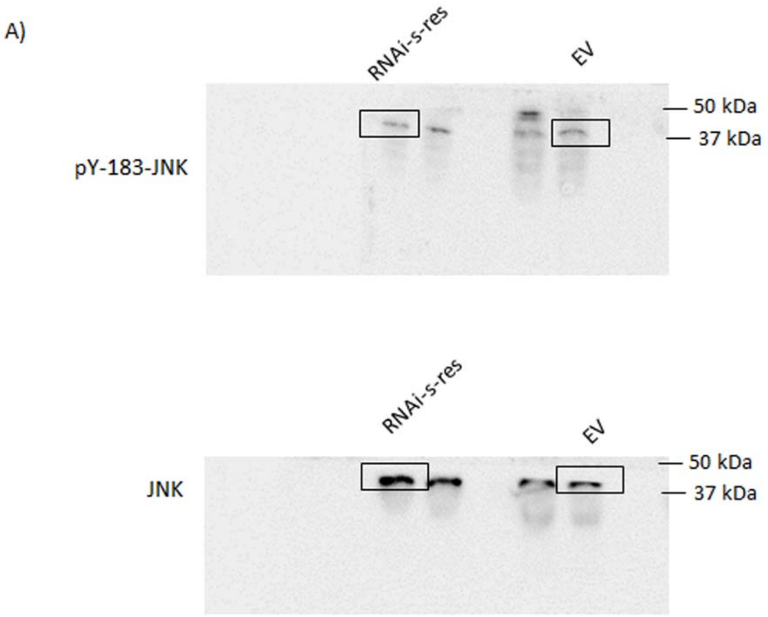

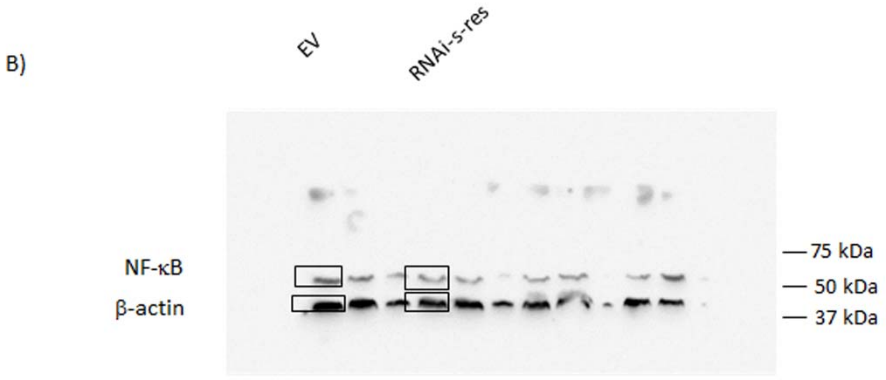

Supplementary Figure S6
